# PinX1-siRNA/mPEG-PEI-SPION combined with doxorubicin enhances the inhibition of glioma growth

**DOI:** 10.3892/etm.2014.1586

**Published:** 2014-02-26

**Authors:** LING LONG, WEIWEI WANG, XIA-DONG CAI, DU CHENG, XINTAO SHUAI, YING PENG

**Affiliations:** 1 Department of Neurology, The Third Affiliated Hospital, Sun Yat-Sen University, Guangzhou, Guangdong 510630, P.R. China; 2 Department of Neurology, Sun Yat-Sen Memorial Hospital, Sun Yat-Sen University, Guangzhou, Guangdong 510120, P.R. China; 3 Center of Biomedical Engineering, School of Chemistry and Chemical Engineering, Sun Yat-Sen University, Guangzhou, Guangdong 510275, P.R. China; 4 Department of Neurology, The Sixth Affiliated Hospital (Guangdong Gastrointestinal and Anal Hospital), Sun Yat-Sen University, Guangzhou, Guangdong 510655, P.R. China

**Keywords:** monomethoxy polyethylene glycol polyethyleneimine superparamagnetic iron oxide nanoparticle, doxorubicin, PIN2-interacting protein 1, short interfering RNA, glioma

## Abstract

Resistance to chemotherapy and the side effects of anticancer drugs are the major obstacles for glioma treatment. The aim of the present study was to develop a novel approach for the treatment of gliomas that improved the therapeutic effect; the anticancer drug, doxorubicin (DOX), was combined with short interfering (si)RNA and monomethoxy polyethylene glycol polyethylenimine superparamagnetic iron oxide nanoparticle (mPEG-PEI-SPION), a magnetic resonance imaging (MRI)-visible nanoparticle. Specific siRNA molecules, delivered by mPEG-PEI-SPION, were employed to knockdown the PIN2-interacting protein 1 (PinX1) gene in C6 glioma cells. PinX1 is a nucleolar protein associated with telomere and telomerase. C6 cells were treated with DOX and/or PinX1-siRNA. The results of the transfection experiments revealed that siRNA/mPEG-PEI-SPION was transfected into C6 cells with high efficiency. PinX1-siRNA was unable to inhibit C6 cells, while in the PinX1-siRNA + DOX group, the same dose of DOX caused an increased loss of cell viability. Therefore, mPEG-PEI-SPION was shown to be viable for siRNA delivery into C6 cells and coadministration of DOX with PinX1-siRNA may be a potential therapeutic method for inhibiting gliomas.

## Introduction

Gliomas are the most common primary malignant tumor of the adult central nervous system, accounting for ~40% of intracranial tumors (1). Gliomas have the worst prognosis due to chemoresistance and radioresistance. Despite multimodality treatments with extensive surgical resection, radiotherapy or chemotherapy, the median survival time of glioma patients is ~1 year (2,3). Therefore, novel strategies are urgently required to increase the survival rate of glioma patients.

The C6 cell line is a rat glioma cell line that is induced by N-nitrosomethylurea. This cell line is widely adopted for glioma study due to the histocompatibility in various categories of rats and the infiltrative growth pattern that is similar to human glioma (4). Therefore, C6 cells were employed in the present study as the cell model for glioma.

Doxorubicin (DOX) is an anticancer agent with a wide spectrum, including breast cancer, lung cancer, leukemia, lymphoma and glioma (5). DOX can penetrate through the cell membrane into cells and combine with chromosomes. The antitumor effects of DOX are exerted mainly through forming a complex with double stranded DNA and strongly interfering with the synthesis of DNA, RNA and also proteins (6). A previous study demonstrated that DOX is able to insert into DNA and cause the splitting of DNA by topoisomerase II. An additional antitumor mechanism of DOX is associated with redox (7). A series of NADPH-dependent cytoreductases reoxidize DOX into semiquinone radicals, which react with oxygen to produce cytotoxic chemicals, including peroxides, hydroxide radicals and hydrogen peroxides. In addition, DOX is hypothesized to combine with lipids on the cell membrane, interfering with a number of cell functions. DOX yields antitumor effects through one or more of the aforementioned mechanisms and cells in all stages of the cell cycle are sensitive to DOX, particularly those in hyperplastic tissues, including tumors. Despite this, the application of DOX is limited due to the side effects, including gastrointestinal adverse reactions, alopecia, allergy, myelosuppression and heart toxicity, among which dose-dependent heart failure is the most significant (8). In addition, specific resistance may occur more easily in single drug therapy. These factors hinder the clinical utility of DOX. However, one method of reducing the side effects and resistance is to use the drug in conjunction with other agents.

Telomeres are nucleoprotein complexes composed of (TTAGGG)^n^ repeats and a specialized protein complex that protect the chromosome-ends from being recognized as deleterious DNA double-stranded breaks, which activates DNA damage responses (9). Telomere length is maintained by telomerase, a specialized reverse transcriptase, that adds TTAGGG repeats to the ends of the chromosomes. In humans and other long-living mammals, telomerase expression is repressed in the majority of somatic cells. As a result, telomeres become increasingly shorter with continuous cell divisions due to the ‘end-replication’ problem (10). This progressive telomere shortening functions as a cell-autonomous barrier against overproliferation, making a potent tumor-suppressor mechanism. The re-expression of telomerase is a key event in tumorigenesis, which is supported by the evidence that telomerase is present in 90% of human cancers (11). The role of telomerase in glioma has also been established, with the rat glioma C6 cell line included. Therefore, telomerase is hypothesized to be a novel and effective target for the therapy of gliomas (12,13).

With fast development of molecular biology and genetics, numerous genes have been identified that are closely associated with tumorigenesis, including a number of oncogenes and tumor suppressor genes. PIN2-interacting protein 1 (PinX1), a nucleolar protein associated with telomere/telomerase, is a putative tumor suppressor. However, the role of PinX1 in telomerase/telomere regulation and cancer remains unclear (14). The expression of the PinX1 mRNA transcript is present in the majority of tested human tumors (15–17). Certain studies consider PinX1 to be an intrinsic telomerase/telomere inhibitor and a putative tumor suppressor, as it binds to and suppresses telomerase enzymatic activity (18). However, other experiments have demonstrated that the depletion of PinX1 expression shortens telomere length and inhibits proliferation in yeast cells (19). In addition, Zhang *et al* demonstrated that silencing PinX1 induces senescence in telomerase-positive cancer cells (20). Therefore, in the present study, PinX1-short interfering (si)RNA was used to downregulate PinX1 mRNA expression and subsequently study the effect on telomerase.

siRNA is rapidly becoming an important tool for gene knockdown and the analysis of gene function (21). Knockdown of specific pathogenic genes is a potent approach for treating diseases, including tumors. In the present study, PinX1 was knocked-down in C6 cells and cell viability was analyzed to confirm the potential of PinX1 as a target gene for the treatment of gliomas. Furthermore, gliomas were treated with a combination of PinX1-siRNA and DOX, with the aim of increasing the efficiency of the treatment and decreasing the side effects.

A major challenge in the study of siRNA is the development of an appropriate delivery system with high efficiency and low toxicity. In previous years, nonviral vectors have become attractive options, among which nanoparticles are suitable candidates for the siRNA delivery system (22). Nanoparticles are a series of structures in the nanometer scale size range (≤100 nm) that retain unique properties. Nanoparticles include polymeric micelles, dendrimers, polymeric and ceramic nanoparticles, protein cage architectures, viral-derived capsid nanoparticles, polyplexes and liposomes (23). As siRNA carriers, nanoparticles have overwhelming superiority since they are biocompatible and biodegradable with strong penetrability and good capacity, but have low toxicity and immunogenecity. Furthermore, by functionalizing the surface with synthetic polymers and appropriate ligands, nanoparticles can be targeted to specific cells and locations within the body or be endowed with specific properties. Superparamagnetic iron oxide nanoparticle (SPION), a highly efficient T2 contrast agent for magnetic resonance imaging (MRI), is widely used experimentally for agent delivery (24). The aforementioned attributes of the SPION-based carrier system enable broad biomedical applications, particularly in *in vivo* studies. In the present study, siRNA molecules with SPION-labeled nanoparticles were delivered to C6 cells to confirm the suitability of this method as a transfection tool. The results of the study may be the foundation for future animal model experiments.

## Materials and methods

### Materials and reagents

Monomethoxy polyethylene glycol [mPEG; molecular weight cut off (MWCO), 2 kDa] and N,N′-carbonyldiimidazole (CDI) were purchased from Sigma-Aldrich (St. Louis, MO, USA). Hyperbranched polyethyleneimine (PEI; MWCO, 25 kDa) was purchased from BASF (Ludwigshafen, Rheinland-Pfalz, Germany). Tetrahydrofuran and chloroform (CHCl_3_) were dried over calcium hydride and distilled prior to use. SPIONs, with an average diameter of 6 nm, were synthesized according to the method reported by Sun *et al* (25). Glioma C6 cells were obtained from the American Type Culture Collection (Rockville, MD, USA) and cell culture media and fetal bovine serum (FBS) were purchased from Invitrogen Life Technologies (Carlsbad, CA, USA). Double-stranded oligonucleotides with homology to a desired target region of PinX1 were synthesized by Guangzhou RiboBio Co., Ltd. (Guangzhou, China) and the target sequence was GCTGTGGATCCCAGAAATA. Cy3-control siRNA and negative control (NC) siRNA were also supplied by Guangzhou RiboBio Co., Ltd. DOX was obtained from Shenzhen Wanle Pharmaceutical Co., Ltd. (Shenzhen, China). Total RNA was extracted with the TRIzol reagent (Invitrogen, Carlsbad, CA, USA). A SYBR PrimeScript quantitative polymerase chain reaction (qPCR) kit and primers were supplied by Takara Biotechnology, Co., Ltd. (Dalian, China). A bicinchoninic acid (BCA) kit was purchased from Pierce Biotechnology, Inc. (Rockford, IL, USA) and a TeloTAGGG Telomerase PCR ELISA kit was purchased from Roche Diagnostics Corporation (Indianapolis, IN, USA). Cell Counting Kit-8 (CCK-8) was obtained from Dojindo (Kumamoto, Japan) and the Hoechst 33243 stain was purchased from Sigma-Aldrich.

### Synthesis of mPEG-PEI-SPION

The mPEG-PEI complex was prepared as previously described (26). The hydroxyl terminal groups of mPEG were activated by CDI in order to allow the conjugation of mPEG to branched PEI. Next, CDI-activated mPEG was conjugated to PEI. The mPEG-PEI-SPION complex was prepared by a ‘ligand exchange’ method as previously reported (27). In total, 200 mg mPEG-PEI and 10 mg SPION were dissolved in 2 ml CHCl_3_. The solution was stirred overnight at room temperature and then precipitated with hexane. The precipitate was dispersed into double distilled water under sonication. Large aggregates were removed by filtering through a 220 nm membrane. The encapsulation efficiency of the SPION was determined using a polarized Zeeman Atomic Absorption Spectrophotometer (Z-2000 series).

### Polyplex siRNA/mPEG-PEI-SPION formation

siRNA and an appropriate amount of mPEG-PEI-SPION (100 nmol siRNA:3.53 μg mPEG-PEI-SPION) (N/P=5) were dissolved separately in double distilled water. The two solutions were mixed by gentle pipetting and the mixture was maintained at room temperature for 30 min to allow polyplex formation.

### ζ-potential and size measurements

The ζ-potential measurements of mPEG-PEI-SPION and siRNA/mPEG-PEI-SPION were conducted using a ZetaPlus instrument (Brookhaven Instruments Corporation, Holtsville, NY, USA) at an angle of 15° at 25°C. The average values plus the SDs were based on the data of three runs. Nanoparticle size was determined using the same instrument at 25°C. Scattering light was detected at a 90° angle and the sizes determined were the mean values of three runs plus the SD.

### Cell culture

The rat glioma C6 cell line was maintained in high glucose Dulbecco’s modified Eagle’s medium, supplemented with 10% FBS and penicillin/streptomycin, at 37°C in a fully humidified atmosphere of 5% CO_2_. When the cells reached confluence, they were trypsinized and subcultured.

### Transfection efficiency of siRNA/mPEG-PEI-SPION

C6 glioma cells were plated in 24-well plates (5×10^4^ cells/well) and allowed to grow overnight. Cy3-siRNA and mPEG-PEI-SPION (at N/P=5) were mixed by gentle pipetting and then incubated for 30 min at room temperature. The original cell culture medium was replaced with medium containing the complexes (concentration of siRNA was 100 nM). The culture was incubated for 4 h at 37°C. After 4 h, the medium was discarded and the cells were washed twice with phosphate-buffered saline (PBS). Next, cells were fixed with paraformaldehyde for 30 min. After washing twice, the cells were stained with 10 μg/ml Hoechst 33243 for 15 min. The cells were observed under a fluorescence microscope (Carl Zeiss AG, Jena, Germany) and fluorescent images were captured and recorded.

### qPCR

C6 cells were plated in 12-well plates (1×10^5^ cells/well) and allowed to grow overnight. Next, the cells were transfected with 100 nM NC-siRNA/mPEG-PEI-SPION or 100 nM PinX1-siRNA/mPEG-PEI-SPION complexes (at N/P=5) as aforementioned. Cells were washed with pre-chilled PBS and collected in TRIzol reagent 48 h following transfection. Total RNA was extracted with the TRIzol reagent according to the instructions provided by the manufacturer. First strand cDNA was synthesized from 1 μg total RNA. The reaction conditions were as follows: 37°C for 15 min and 85°C for 5 sec. PCR was performed in a 20 μl volume (1 μl cDNA) with SYBR green dye. Sequence-specific oligonucleotide primers were as follows: PinX1, 5′-AACCACCTGGGACTTGGAGCTA-3′ (forward) and 5′-CCTGACCATGGCAAGTGTTGA-3′ (reverse); and β-actin, 5′-GGAGATTACTGCCCTGGCTCCTA-3′ (forward) and 5′-GACTCATCGTACTCCTGCTTGCTG-3′ (reverse). The reaction conditions were as follows: 95°C for 30 sec, followed by 40 cycles of 95°C for 5 sec and 60°C for 34 sec. β-actin was amplified as the housekeeping gene and qPCR assays of all the samples were performed in triplicate.

### Telomerase activity assay

Telomerase activity was measured with a TeloTAGGG Telomerase PCR ELISA kit. C6 cells were transfected with NC-siRNA/mPEG-PEI-SPION or PinX1-siRNA/mPEG-PEI-SPION as aforementioned. After 48 h, cellular extracts were prepared with 1X CHAPS lysis buffer. The protein concentration was determined using a BCA kit. An extract equal to 1 μg protein was amplified with the reaction mixture included in the kit. The extract and reaction mixture were maintained at 25°C for 30 min, then heated at 90°C for 3 min and subjected to 30 cycles of PCR with specific programing (94°C for 30 sec, 50°C for 30 sec and 72°C for 90 sec). Following an additional 10 min at 72°C, the amplification products were subjected to hybridization and ELISA, according to the instructions provided by the manufacturer. The absorbance value of each sample was calculated using a microplate reader at 450 nm, with a reference of 690 nm. Each sample was tested in triplicate.

### Cell viability

For the CCK-8 test, C6 cells were plated in 96-well plates at an initial density of 1×10^4^ cells/well. PinX1-siRNA/mPEG-PEI-SPION and NC-siRNA/mPEG-PEI-SPION (at N/P=5) complexes were constructed as aforementioned. The original cell culture medium was replaced with medium containing one of the complexes (100 nM siRNA) and the cells were incubated for 4 h at 37°C. After 4 h, the medium was replaced with fresh complete medium, with or without 10 μg/ml DOX, and cells were allowed to grow for 20 h. After 24 h of DOX incubation, the CCK-8 reagent was added to the wells and incubated at 37°C for 1 h. Absorbance at 570 nm of each well was recorded with the microplate reader. Each treatment group was replicated in three wells.

### Cell apoptosis

C6 cells were plated in 24-well plates (5×10^4^ cells/well) and allowed to grow overnight. Transfection and DOX incubation were performed as aforementioned. Following DOX incubation for 24 h, the medium was discarded and the cells were washed with PBS three times. Next, cells were fixed with paraformaldehyde for 30 min and washed twice. The cells were stained with 10 μg/ml Hoechst 33243 for 15 min and a fluorescence microscope was used for observation.

### Statistical analysis

Results were analyzed using SPSS version 12.0 (SPSS, Inc., Chicago, IL, USA) and compared using one way analysis of variance with Fisher’s least significant difference post hoc test. Data are presented as the mean ± SD and P<0.05 was considered to indicate a statistically significant difference.

## Results

### Size and ζ-potential of the nanoparticles

The average size and ζ-potential of the mPEG-PEI-SPION and siRNA/mPEG-PEI-SPION complexes are shown in Table I. Sizes of PinX1-siRNA/mPEG-PEI-SPIONs ranged between 100 and 150 nm, which was a suitable size for higher specific surface area and better penetrability. The average ζ-potential of PinX1-siRNA/mPEG-PEI-SPIONs was 25.27±1.75 mV, which enabled the absorption of PinX1-siRNA/mPEG-PEI-SPIONs through the cell membrane which has a negative potential.

### Transfection and knockdown

**Figure 1:**
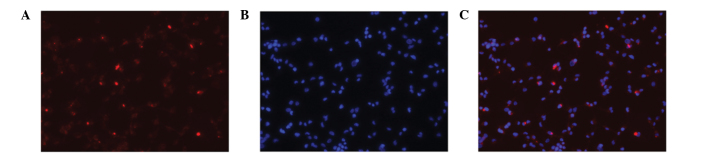
Fluorescent images of C6 cells after 4 h incubation with Cy3-siRNA/mPEG-PEI-SPION in (A) Cy3-contained cells, (B) Hoechst stained cells and (C) a merged image. As demonstrated in the images, Cy3 was detected in the cytoplasm of almost all the cells, indicating that Cy3-siRNA/mPEG-PEI-SPION was transfected with high efficiency. mPEG, monomethoxy polyethylene glycol; PEI, polyethyleneimine; SPION, superparamagnetic iron oxide nanoparticle; siRNA, short interfering RNA.

**Figure 2:**
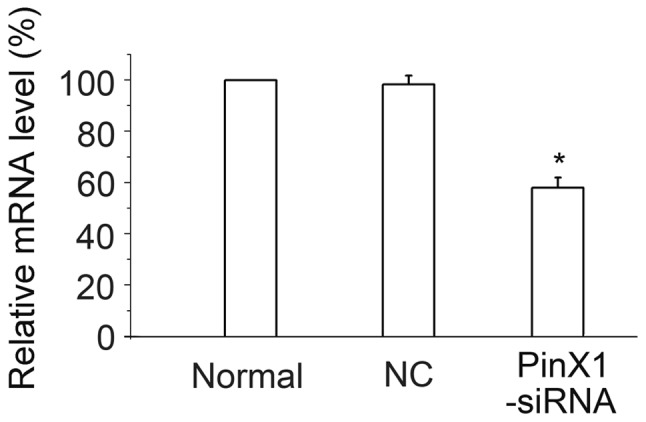
qPCR analysis of PinX1 mRNA expression levels revealed that NC-siRNA did not affect the PinX1 mRNA expression levels (P>0.05, vs. normal group). However, PinX1-siRNA did reduce the PinX1 mRNA expression levels to 57.7% of the NC group (^*^P<0.05, vs. normal group). qPCR, quantitative polymerase chain reaction; PinX1, PIN2-interacting protein 1; siRNA, short interfering RNA; NC, negative control.

**Figure 3:**
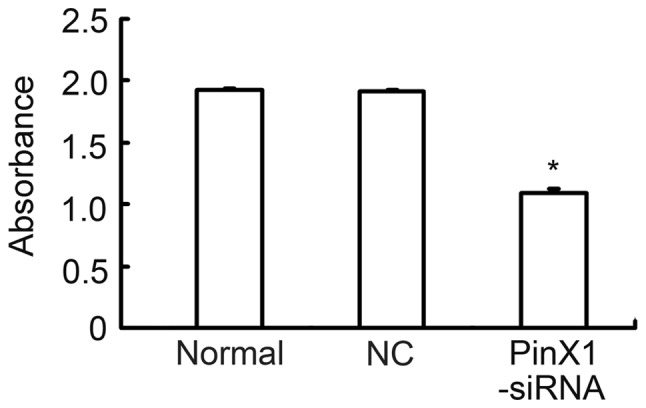
Absorbance values of each group using the telomerase PCR ELISA assay. There was no significant difference in the absorbance values between the NC-siRNA and normal groups. However, the PinX1-siRNA group exhibited a markedly decreased absorbance value as compared with the normal group. ^*^P<0.05, vs. normal group. PCR, polymerase chain reaction; PinX1, PIN2-interacting protein 1; siRNA, short interfering RNA; NC, negative control.

In the transfection of Cy3-siRNA/mPEG-PEI-SPION into C6 cells, Cy3-siRNA/mPEG-PEI-SPION was shown to be efficiently delivered into C6 cells. As shown in Fig. 1, almost all the C6 cells were transfected with Cy3-siRNA/mPEG-PEI-SPION. The following qPCR experiment demonstrated that PinX1-siRNA/mPEG-PEI-SPION transfection resulted in a knockdown of PinX1 mRNA expression (Fig. 2), while NC-siRNA/mPEG-PEI-SPION did not have this effect. Therefore, mPEG-PEI-SPION is an ideal tool for delivering siRNA into C6 cells. Furthermore, transfection of 100 nM PinX1-siRNA/mPEG-PEI-SPION significantly decreased telomerase activity in C6 cells (Fig. 3).

### Cell viability

**Figure 4:**
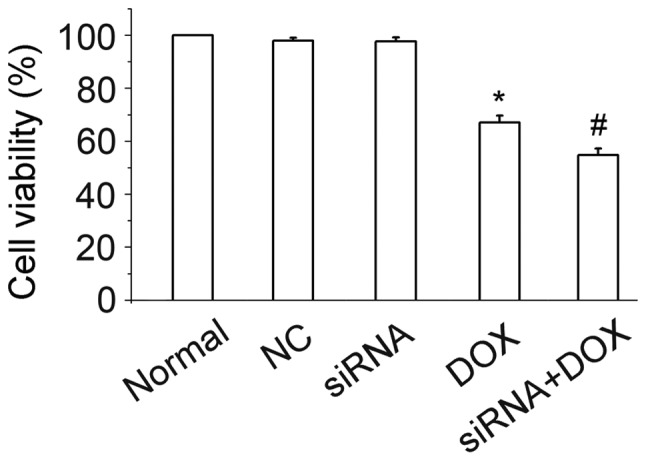
PinX1-siRNA increased the cell toxicity of DOX. Treatment with 10 μg/ml DOX reduced cell viability to 67% of the normal group. PinX1-siRNA alone did not decrease the cell viability. Cells treated with a combination of PinX1-siRNA and DOX exhibited a cell viability of 54.7%, which was significantly lower when compared with the DOX group. ^*^P<0.05, vs. normal group; ^#^P<0.05, vs. DOX group. DOX, doxorubicin; PinX1, PIN2-interacting protein 1; siRNA, short interfering RNA.

**Figure 5:**
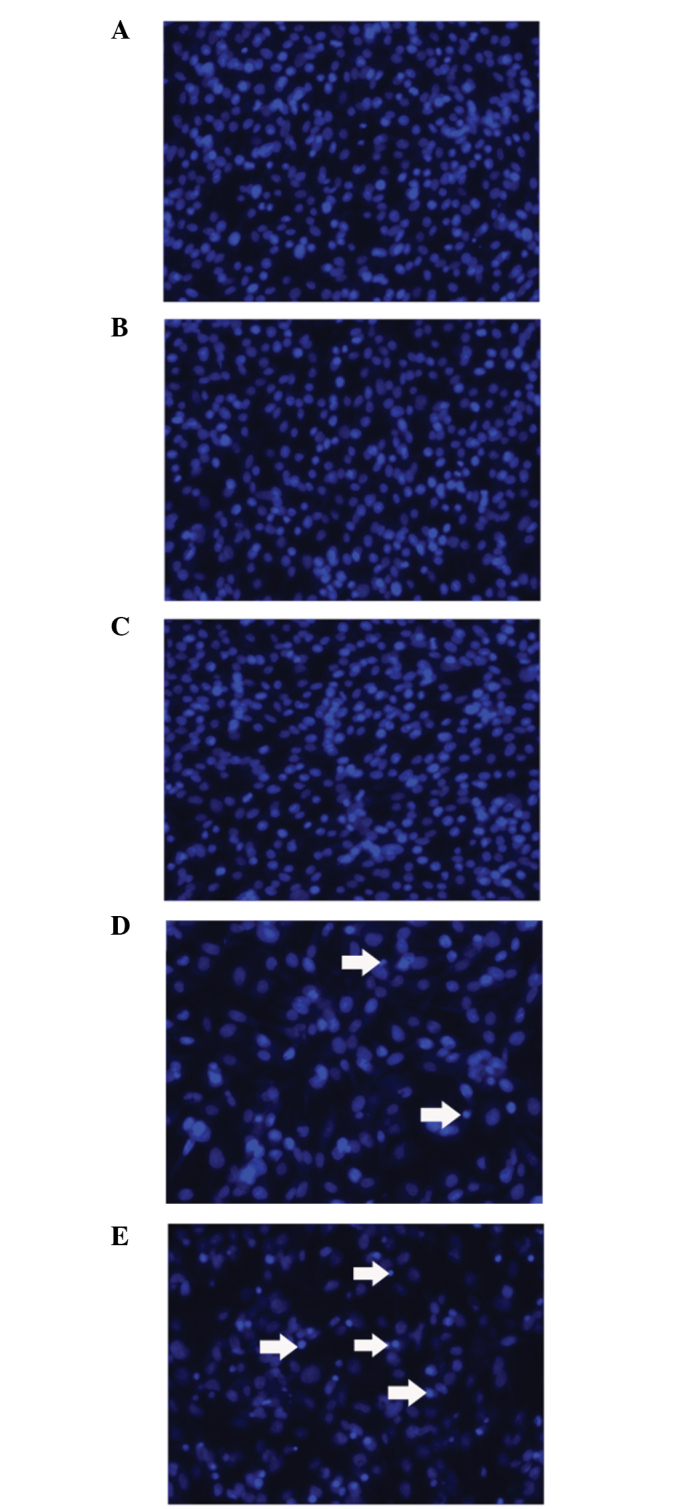
Hoechst staining images of C6 cells in the (A) normal control, (B) NC-siRNA control, (C) PinX1-siRNA, (D) DOX and (E) DOX + PinX1-siRNA groups. Representative images revealed that 10 μg/ml DOX caused decreased cell numbers and nucleus fragmentation (arrows). Furthermore, if C6 cells were administered with PinX1-siRNA and DOX simultaneously, cell loss and nuclear condensation (arrows) were markedly more severe. DOX, doxorubicin; PinX1, PIN2-interacting protein 1; siRNA, short interfering RNA; NC, negative control.

Administration of 10 μg/ml DOX resulted in a prominent decrease in cell viability when compared with the normal controls. PinX1-siRNA alone was unable to cause cytotoxicity in the observed time period, however, when used in combination with DOX, PinX1-siRNA was able to sensitize the inhibition effect of DOX. PinX1-siRNA decreased cell viability by ~12.3%, as measured by the CCK-8 method (Fig. 4). In addition, PinX1-siRNA enhanced the cell toxicity of DOX by promoting cell death and apoptosis, as shown by Hoechst 33243 staining (Fig. 5).

## Discussion

The anthracycline chemical DOX is a common antitumor agent, however, the drug is limited by its toxicity to healthy organisms, of which acute or chronic heart failure is the most severe (28).

Although siRNA has been shown to be a promising therapy, the delivery system is one of the obstacles for siRNA application, particularly in the nervous system which is difficult to transfect. Viruses mediate transfection at a high efficiency, but are limited by their immunogenicity. Nanomedicine is a rapidly developing field, and due to the increasing interest, marked progress has been made in the medical applications of nanoscale devices. Nanoparticles have been applied in disease diagnosis, treatment and prevention. Among them, cationic polymers are the most common. PEG, a type of cationic polymer, has been increasingly studied and identified to have a high transfection efficiency, thus, currently PEG is considered to be the gold standard of transfection efficiency (29). Furthermore, PEG has been modified with PEI to obtain mPEG-PEI, an improved nanoparticle with less toxicity, more target activity and improved stability. In addition, mPEG-PEI can be labeled with other molecules to obtain various characteristics, including SPION, a magnetic nanoparticle which is detectable by MRI. Medarova *et al* (30) successfully used SPION-labeled nanoparticles to deliver siRNA to tumor cells. In the present study, mPEG-PEI-SPION was competent in forming complexes with siRNA and entering C6 cells, which facilitated further *in vivo* study with MRI. To the best of our knowledge, the current study is the first to apply this system in C6 cells.

Telomeres function as protective structures that cap the ends of chromosomes. They consist of terminal TTAGGG repeats and telomere-specific DNA binding proteins. With each cell division, the 5′ end of the telomere is shortened by 50–200 nucleotides. Therefore, following several replications, the telomeres reach a threshold and cell proliferation arrest or cell death occurs. Telomerase is an RNA-dependent DNA polymerase that functions as a reverse transcriptase which is responsible for the synthesis of telomeres. Telomerase prevents the shortening of telomeres and is essential for the maintenance of telomere length and activity. The activation of telomerase is considered to be critical in cell immortalization (11) and increasing evidence indicates that telomere dysfunction is a common driver for the genomic instability that is present in cancer (31). Ding *et al* reactivated telomerase expression with the inducible mouse telomerase reverse transcriptase transgene in a prostate cancer mouse model. The authors found that re-expression of telomerase in the prostate epithelium generated more aggressive tumors (32). These results further indicate that telomerase may be a potent target for combating tumors.

PinX1 is a conserved nucleolar protein that has complex roles in telomerase/telomere regulation and cancer. In the study by Zhou and Lu, downregulation of PinX1 expression via antisense cDNA transfection resulted in increased telomerase activity, increased telomere length and enhanced tumor malignancy in the HT1080 (telomerase-positive) cancer cell line. These results indicated that PinX1 is a telomerase/telomere inhibitor and a putative tumor suppressor in humans (18). However, whether PinX1 is tumor-suppresive or promotive remains elusive. In the present study, transfection of PinX1-siRNA/mPEG-PEI-SPION into C6 cells not only resulted in the significant downregulation of PinX1 mRNA expression, but transfection also weakened telomerase activity. These observations support the hypothesis that PinX1 may be a target for suppressing tumor growth, in accordance with the study by Zhang *et al* (20).

The aims of the present study were to determine whether mPEG-PEI-SPION may be a device for siRNA delivery into C6 cells and whether the specific silencing of PinX1 by siRNA may improve the cytotoxic effect of DOX in C6 glioma cells. As demonstrated, a combination of PinX1-siRNA/mPEG-PEI-SPION and DOX resulted in increased cell loss when compared with DOX administration alone. Therefore, PinX1-siRNA/DOX is more efficient for inhibiting C6 growth. The results of the present study support the hypothesis that combined therapy with PinX1-siRNA/DOX can successfully maintain the tumor inhibition effect with reduced side effects.

In conclusion, the current study has demonstrated that mPEG-PEI-SPION is viable in delivering siRNA to C6 cells for the purpose of treatment. Furthermore, PinX1 may regulate telomerase activity and is therefore a potential target for inhibiting tumors. To the best of our knowledge, this is the first study to treat gliomas with a combination of DOX and PinX1-siRNA. In addition, since SPIONs can be monitored by MRI, the present study may be used as a pilot study for further investigation into the application of siRNA/mPEG-PEI-SPION for brain tumors *in vivo*.

## Figures and Tables

**Table I:** ζ-potential and size of mPEG-PEI-SPION and PinX1-siRNA/mPEG-PEI-SPION (N/P=5).

Nanoparticle	ζ-potential, mV	Size, nm
mPEG-PEI-SPION	34.42±0.78	39.6±1.2
PinX1-siRNA/mPEG-PEI-SPION (N/P=5)	25.27±1.75	126.3±2.3

mPEG, monomethoxy polyethylene glycol; PEI, polyethyleneimine; SPION, superparamagnetic iron oxide nanoparticle; siRNA, short interfering RNA; PinX1, PIN2-interacting protein 1.
